# Supplementary material for: First-principles calculation of adsorption of cadmium and lead on the surface of a 1T-MnO2 monolayer
Source: PLoS One. 2025 Nov 14;20(11):e0335153. doi: 10.1371/journal.pone.0335153 (PMC12617903; doi:10.1371/journal.pone.0335153)
Supplement: Attachment — Submitted filename: Comments.docx [file pone.0335153.s001.docx]

**First-principles calculation of adsorption Cd and Pb on the surface of a 1T-MnO2 monolayer**

**Comments**

**Abstract:**

1. the charge transfer in the adsorbate/monolayer interaction 1T-MnO2 is established via the Bader charge. Please correct “the” to “The”.
2. Please avoid using abbreviations in the keyword and make it short too.
3. The MnO2 monolayer crystallizes in the trigonal (denoted 1H) phase with the D3h point group and the octahedral (denoted 1T) with D3d point group. Please explain D3h and D3d point groups.
4. The introduction needs to be improved. It is too short and includes examples of computational studies.
5. Authors are advised to include the figure just above the figure caption for better understanding.
6. **Figure 4.** Contour map of the charge distribution on the T-MnO2 monolayer, side view. The red color indicates little or no charge, and blue indicates high charge presence. The atoms of the upper and lower plane are of oxygen, and those of the central plane are of manganese. Please increase the font size of Figure 4.

Overall comment: The manuscript is well written and explained. However, some changes are required in the introduction section and the grammar of the manuscript needs to be checked once again. The manuscript can be published if all the comments are resolved.
