# Supplementary material for: First-principles calculation of adsorption of cadmium and lead on the surface of a 1T-MnO2 monolayer
Source: PLoS One. 2025 Nov 14;20(11):e0335153. doi: 10.1371/journal.pone.0335153 (PMC12617903; doi:10.1371/journal.pone.0335153)
Supplement: Attachment — Submitted filename: Comments_declet_0.docx [file pone.0335153.s002.docx]

**Comments**

**The manuscript titled ‘First-principles calculation of adsorption Cd and Pb on the surface of a 1T-MnO₂ monolayer’ is well-structured and presents an interesting approach. However, several aspects require significant improvement. I recommend revisions to enhance its clarity, consistency, and overall impact.**

- The abstract outlines a relevant and technically valuable study; however, it requires major revision to improve clarity, fix grammatical errors, and correct awkward phrasing such as “adsorption the toxic heavy atoms,” as well as repeated wording like “of the of the.”
- Terms like PMn and others must be clearly defined at their first use throughout the manuscript.
- Correct typographical errors, such as changing “Quatum-Espresso” to “Quantum ESPRESSO,” to ensure accuracy and consistency throughout the manuscript.
- “T-MnO₂” and “1T-MnO₂” are used interchangeably without clarification.
- The conclusion section should be revised to be more concise.
