# Supplementary material for: First-principles calculation of adsorption of cadmium and lead on the surface of a 1T-MnO2 monolayer
Source: PLoS One. 2025 Nov 14;20(11):e0335153. doi: 10.1371/journal.pone.0335153 (PMC12617903; doi:10.1371/journal.pone.0335153)
Supplement: Attachment — Submitted filename: response reviewers.docx [file pone.0335153.s003.docx]

**RESPONSE REVIEWERS**

Dear editor PLOS ONE Journal

We have made the suggested corrections, which are marked in red in the manuscript.

Editor and Reviewer comments:

**Reviewer 1**

**Comments**

**The manuscript titled ‘First-principles calculation of adsorption Cd and Pb on the surface of a 1T-MnO₂ monolayer’ is well-structured and presents an interesting approach. However, several aspects require significant improvement. I recommend revisions to enhance its clarity, consistency, and overall impact.**

1. The abstract outlines a relevant and technically valuable study; however, it requires major revision to improve clarity, fix grammatical errors, and correct awkward phrasing such as “adsorption the toxic heavy atoms,” as well as repeated wording like “of the of the.”

R/ We have made the corrections.

2. Terms like PMn and others must be clearly defined at their first use throughout the manuscript.

R/ The terms PMn and others are defined in section 3.2.1. in red text.

To determine the most favorable adsorption models of Cd and Pb onto the 1T-MnO_2_-3x3 monolayer, we considered five (5) non-equivalent special sites, above an oxygen atom O ( site P_O1_): in the upper plane oxigen atoms; above a manganese atom Mn (site P_Mn_): in the intermediate plane of manganese atoms; above an oxygen atom O (site P_O2_): in the lower plane, and above the Br_1_ bridge (site P_B1_), at the midpoint between an oxygen atom of the upper plane and an oxygen atom of the lower plane, and above Br_2_ bridge (site P_B2_): at the midpoint between an oxygen atom of the upper plane and a manganesse atom of the intermediate plane, as shown in Figure 6.

3. Correct typographical errors, such as changing “Quatum-Espresso” to “Quantum ESPRESSO,” to ensure accuracy and consistency throughout the manuscript.

**R/** We have made the corrections

4. “T-MnO₂” and “1T-MnO₂” are used interchangeably without clarification.

R/ We would like to express our embarrassment for having made a notation error, since the correct notation is 1T-MnO₂. We have made the corrections.

5. The conclusion section should be revised to be more concise.

R/ The conclusion is now:

In summary, the structural and electronic properties, the stability, and the Cd and Pb adsorption on the 1T-MnO2 monolayer were studied using first-principles calculations. We found that the P_Mn_ site is the most energetically favorable for the adsorption of a Cd or Pb atom, with adsorption energies of – 0.883 eV and -5.918 eV, respectively. The Cd atom transfers a charge of 1e^-^ to the monolayer, and the Pb transfers 1.4e. Aditionally, once the adsorption is complete, the Cd and the Pb atoms are bound to the monolayer by means of an ionic bond.

In relation to the pristine monolayer, the calculated of the exfoliation energy is 16,925 meV/A, from which it can be deduced that the monolayer is easily exfoliable. The calculations the energy formation (Ef = -5.741 eV), the elastic constant and vibrational properties show that the monolayer is thermodynamic, mechanic and vibrationally stable. The monolayer exhibits semiconductor behavior, with indirect band gaps of 2.08 eV and 2.02 eV for the spin-up and spin-down components, respectively. Furthermore, it exhibits magnetic behavior, with a magnetic moment of 3μ_β_/cell.

**Reviewer 2**

**Comments**

**Abstract:**

1. the charge transfer in the adsorbate/monolayer interaction 1T-MnO2 is established via the Bader charge. Please correct “the” to “The”.

**R/** We have made the corrections

2. Please avoid using abbreviations in the keyword and make it short too.

**R/** We have made the corrections

3. The MnO2 monolayer crystallizes in the trigonal (denoted 1H) phase with the D3h point group and the octahedral (denoted 1T) with D3d point group. Please explain D3h and D3d point groups.

R/ In the introduction section, we have added a brief description to explain D3h and D3d point groups, and we have added the text in red:

The MnO_2_ monolayer crystallizes in the trigonal (denoted 1H) phase with D_3h_ point group (in the Hermann-Mauguin notation, D_3h_ = $\bar{6}$m2, where $\bar{6}$is called the rotation-inversion axis (improper rotation) and consists of a rotation of order 6 (2π/6 rad=π/3 rad=60°) followed by a $\bar{1}$inversion equivalent to a plane of symmetry or mirror, m is the vertical plane of symmetry (parallel to the main axis), and 2 the rotation axis of order 2 (2π/2 rad=π rad=180° perpendicular to the principal axis), and the octahedral (denoted 1T) with D_3d_ point group (in the Hermann-Mauguin notation, D_3d_ = $\bar{3}$m, where $\bar{3}$is called the rotation-inversion axis (improper rotation) and consists of a rotation of order 3 (2𝜋/3𝑟𝑎𝑑=120°) followed by an inversion $\bar{1}$ (equivalent to a plane of symmetry or mirror); m is the symmetry plane (diagonal)).

4. The introduction needs to be improved. It is too short and includes examples of computational studies.

R/ We have added more computational studies, and five more references related to theoretical studies. In the paragraph we added, the text is in red.

Recent experimental studies reveal that the manganese dioxide monolayer is an excellent material for the removal of atoms, molecules, and gases that are very dangerous global pollutants due to their high toxicity. Wu *et al.* [21] demonstrated that sulfur dioxide (SO_2_) is efficiently captured via heterogeneous oxidation into sulfate on the surface of manganese dioxide (MnO_2_) with an efficiency in the capture of nearly 100%. Liu *et al.* [22] developed a filter to capture SO_2_ produced in the combustion of fossil fuels and released into the environment by vehicles and industrial smokestacks. Peng *et al.* [23] demonstrated the great potential of the MnO_2_ monolayer for the electrocatalytic transformation of CO_2_ to valuable chemicals products, because they proved that manganese dioxide reduces CO_2_ to CO with an efficiency of 71%. This is great importance for the chemical industry, because it constitutes an alternative path for the synthesis of important chemical feedstocks and complex carbon-based fuels. On the other hand, theoretical studies predict the use of the MnO_2_ monolayer in different areas. Deng *et al.* [24], using first-principles calculations, showed that it exhibits good performance for Li storage capacity and diffusion, and therefore the monolayer is promising electrode material for high-capacity Li ion batteries. Chen *et al.* [25], using density functional calculations, studied the adsorption of 5-fluorouracil (5-FU) on the surface of MnO2 and predicted that the monolayer is a good material for targeted delivery of drug molecules hiking on nanomaterials. Additionally, other theoretical studies based on the framework of density functional theory have focused on the adsorption of toxic atoms and molecules that cause environmental pollution. Independently, Zhu *et al.* [26] and Li *et al.* [27] studied the adsorption of NO and O_2_ molecules on the MnO_2_ surface, finding that this material is an excellent candidate for reducing emissions of these gases. Zhen *at al.* [28] calculated the adsorption of elemental mercury (Hg^0^) on MnO_2_; meanwhile, Zhang *et al.* [29] studied the adsorption mechanism of mercury species (HgO, HgCl, and HgCl_2_) on the MnO_2_ surface. These theoretical investigations predicted that MnO_2_ is a good material for the adsorption and oxidation of the Hg^0^ and mercury species HgO, HgCl, and HgCl_2_, and therefore MnO_2_ is a very promising material for the construction of devices (filters) that allow reducing emissions of mercury and mercury species to the environment that result from the burning of fossil fuels from power plants and vehicle exhausts [30].

5. Authors are advised to include the figure just above the figure caption for better understanding.

R/ We have included the figures in the manuscript.

6. **Figure 4.** Contour map of the charge distribution on the T-MnO2 monolayer, side view. The red color indicates little or no charge, and blue indicates high charge presence. The atoms of the upper and lower plane are of oxygen, and those of the central plane are of manganese. Please increase the font size of Figure 4.

R/ Thank you very much for the suggestion. We have increased the size of Figure 4.

Overall comment: The manuscript is well written and explained. However, some changes are required in the introduction section and the grammar of the manuscript needs to be checked once again. The manuscript can be published if all the comments are resolved.
