# Supplementary material for: First-principles calculation of adsorption of cadmium and lead on the surface of a 1T-MnO2 monolayer
Source: PLoS One. 2025 Nov 14;20(11):e0335153. doi: 10.1371/journal.pone.0335153 (PMC12617903; doi:10.1371/journal.pone.0335153)
Supplement: Attachment — Submitted filename: response_reviewers_auresp_2.docx [file pone.0335153.s004.docx]

**RESPONSE REVIEWERS**

Editor, *PLOS ONE Journal*

Dear sir:

We have made the suggested corrections, which are marked in red in the manuscript.

Editor and Reviewer comments:

Journal Requirements, editor and Reviewer comments:

Journal Requirements:

1. If the reviewer comments include a recommendation to cite specific previously published works, please review and evaluate these publications to determine whether they are relevant and should be cited. There is no requirement to cite these works unless the editor has indicated otherwise.

R/ Reviewer #3 suggests including three references, but we do not believe they are relevant for the article, since we did not find that they are germane to the material and the atoms that are absorbed in this investigation.

2. Please review your reference list to ensure that it is complete and correct. If you have cited papers that have been retracted, please include the rationale for doing so in the manuscript text, or remove these references and replace them with relevant current references. Any changes to the reference list should be mentioned in the rebuttal letter that accompanies your revised manuscript. If you need to cite a retracted article, indicate the article’s retracted status in the References list and also include a citation and full reference for the retraction notice.

R/ We have reviewed the list of references, and all 55 references in the list are cited in the article. None of the cited articles have been retracted, as verified by the DOI of each reference.

**Editor Comments:**

Dear Author,

The manuscript titled "First-principles calculation of adsorption Cd and Pb on the surface of a 1T-MnO2 monolayer” is found it to be interested and informative work. However, I have few suggestions for minor revisions that I believe will further enhance the clarity and impact of your manuscript:

1. Please do not bold equations mentioned in the manuscript (e.g. 3.1, 3.2……..).

R/ We have made the corrections.

2. Please make sure to define each acronym at its first use. Check through the entire manuscript to make sure it is defined at the first use.

R/ We have reviewed all of the acronyms and have defined them the first time they are used. In addition, we have defined the stoichiometric formulas of HgO, HgCl, and HgCl_2_ before using them.

3. Grammar and Punctuation: There are several grammatical and punctuation errors throughout the manuscript.

R/ We have had a careful review done and have corrected the errors.

4. Please add limitations of study and future direction as separate section in the manuscript. Please do not use unrelated citations suggested by reviewers.

R/ We have added a section before the conclusions titled “**Limitations and perspectives**”.

**Reviewer Comments**

**1. If the authors have adequately addressed your comments raised in a previous round of review and you feel that this manuscript is now acceptable for publication, you may indicate that here to bypass the “Comments to the Author” section, enter your conflict of interest statement in the “Confidential to Editor” section, and submit your "Accept" recommendation.**

**Reviewer #1: All comments have been addressed**

**Reviewer #2: All comments have been addressed**

**Reviewer #3: All comments have been addressed**

**R/ Thank you very much for your comments.**

**2. Is the manuscript technically sound, and do the data support the conclusions?**

**The manuscript must describe a technically sound piece of scientific research with data that supports the conclusions. Experiments must have been conducted rigorously, with appropriate controls, replication, and sample sizes. The conclusions must be drawn appropriately based on the data presented.**

**Reviewer #1: Yes**

**Reviewer #2: Yes**

**Reviewer #3: Yes**

**R/ Thank you very much for your comments.**

**3. Has the statistical analysis been performed appropriately and rigorously?**

**Reviewer #1: Yes**

**Reviewer #2: Yes**

**Reviewer #3: Yes**

**R/ Thank you very much for your comments.**

**4. Have the authors made all data underlying the findings in their manuscript fully available?**

**The PLOS Data policy requires authors to make all data underlying the findings described in their manuscript fully available without restriction, with rare exception (please refer to the Data Availability Statement in the manuscript PDF file). The data should be provided as part of the manuscript or its supporting information, or deposited to a public repository. For example, in addition to summary statistics, the data points behind means, medians and variance measures should be available. If there are restrictions on publicly sharing data—e.g. participant privacy or use of data from a third party—those must be specified.**

**Reviewer #1: Yes**

**Reviewer #2: Yes**

**Reviewer #3: Yes**

**R/ Thank you very much for your comments.**

**5. Is the manuscript presented in an intelligible fashion and written in standard English?**

**PLOS ONE does not copyedit accepted manuscripts, so the language in submitted articles must be clear, correct, and unambiguous. Any typographical or grammatical errors should be corrected at revision, so please note any specific errors here.**

**Reviewer #1: Yes**

**Reviewer #2: No**

**Reviewer #3: Yes**

**R/ Thanks to reviewers 1 and 3 for their positive comments.**

**Reviewer 2: We have had a complete review of the manuscript done in order to correct all errors.**

**6. Review Comments to the Author**

**Please use the space provided to explain your answers to the questions above. You may also include additional comments for the author, including concerns about dual publication, research ethics, or publication ethics. (Please upload your review as an attachment if it exceeds 20,000 characters)**

**Reviewer #1: Authors have addressed all the comments in a well-mannered way and followed all the guidelines of the journal. Now it can be published in PLOS one.**

**Reviewer #2: The authors have thoroughly addressed all previously raised concerns, including clarifications in terminology, grammatical corrections, and improvements in technical writing.**

**Reviewer #3: The authors did not included the references I have selected, and therefore please review all of my comments address them individually**

**R/ Thanks to reviewers 1 and 2 for their positive comments.**

With respect to reviewer #3’s comment, we are embarrassed, because in the former response to the reviewers, we did not attach the responses to reviewer 3.

**Responses to reviewer #3's comments:**

**Reviewer #3:**

**Regarding Computational Methodology:**

1. Could the authors provide more details about the specific exchange-correlation functional used within DFT? Different functionals can influence the calculated adsorption energies and charge transfer. A justification for the chosen functional's suitability for this system would be beneficial.

R/ In our investigation, we opted for the GGA-PBE approximation with ultrasoft pseudopotentials (USPP), which offers a balance between computational precision and computational efficiency. This choice was based on the critical analysis by Stroppa et al. [1], in which he establishes how hybrid functionals (B3LYP and BLYP) “fail for metals and energy of adsorption” with unacceptable errors up to 50% in atomization energy and 10% in atomic systems for metals with d orbitals, thus invalidating their use in adsorption, since “it does not make sense to predict surface properties if the substrate is not described with precision”. Other hybrid functionals, such as HSE (Heyd-Scuseria-Ernzerhof) and PBE0, although they considerably improve the precision of the electronic calculations and the band structure, they carry a high computational cost due to the inclusion of an exact Hartree-Fock interchange fraction. However, studies such as that by Capdevila et al. [2] demonstrate that these high-precision results can be replicated efficiently by means of traditional DFT+U methods, as long as an adequate value for the Hubbard parameter (U) is selected. In their study, they establish that the GGA+U, with U within a eV range [3,4], for manganese (Mn), allows reaching results similar to those obtained using hybrid functionals and other experimental results. In particular, in the present investigation a Hubbard parameter of 3.9 eV was used, which is within the aforementioned range.

With regard to semi-local alternatives, there exists a paradox: functionals such as AM05/PBEsol describe systems in volume well, but overestimate the values of the results in the adsorption processes, while rPBE/BLYP underestimate the cohesion in solids but predict the values of the results of the processes of adsorption well. Stroppa et al. [1] emphasize that no semi-local functional is able to resolve both problems simultaneously, proposing the PBE approximation as a practical option, since it avoids catastrophic errors in the metallic substrate and offers an adequate balance between the properties of the material and the processes pertaining to adsorption. Likewise, this choice of the PBE functional with USPP (ultrasoft) pseudopotentials instead of PAW (projected augmented waves) is justified after critically evaluating the precision-efficiency compensation. Although PAW may offer marginal improvements in electronic properties on dealing with valance electrons with greater rigor, its computational cost is significantly increased in extended systems such as surfaces and adsorption processes.

Finally, the functionals of the SCAN, SCAN, rSCAN and r²SCAN family present a paradox similar to that of the hybrid methods: while they achieve exceptional precision in structural properties like lattice constants and optimal geometries, their implementation carries an undesirable computational cost for extended systems. Swathilakshmi et al. [3] showed evidence of this dichotomy when evaluating diverse SCAN functionals in transition metal oxides (TMOs) Their study reveals that in spite of its structural precision, these functionals exhibit critical deficiencies in electronic description: they frequently predict spurious metallicity in semiconductor systems and systematically distort (significant underestimation or overestimation) the bandgaps.

Given our prioritization of the viability of large-scale calculations (essential for modelling complex interfaces), the marginal gain in precision offered by costly functionals such as B3LYP, HSE, and PBE0 hybrids or meta-GGA such as SCAN does not justify their use, due to the operative cost overrun. This decision is reinforced by the analysis by Stroppa et al. [1], who point out that the intrinsic limitations of the PBE functional, such as systematic errors in bandgaps, dominate over technical variations between pseudopotentials. As a consequence, the choice of ultrasoft pseudopotentials (USSP) preserves the physical quality inherent in PBE for metallic systems, optimizing the balance between the fundamental rigor and the computational agility required for exploring extended interfacial phenomena.

2. What was the size of the 1T-MnO2 monolayer used in the calculations, and were any convergence tests performed to ensure that the size of the monolayer was sufficient to accurately model the adsorption of Cd and Pb? Providing information about the supercell size and k-point sampling is crucial.

R/ In the second line of the computational methodology, the size of the monolayer is given as 3x3. Additionally, an 8x8x1 mesh of k-points was used.

The number of K points was chosen after constructing a convergence curve.

3. How were van der Waals (vdW) interactions accounted for in the calculations? vdW forces can play a significant role in the adsorption of heavy metals on surfaces, and their inclusion (or lack thereof) should be clearly stated and justified.

R/ We forgot to include that information; we have corrected the mistake and added:

The Van de Waals interactions between the heavy metals (Cd and Pb) and the monolayer are considered using the semiempirical Grimme DFT-D2 correction [38].

4. Were spin-polarized calculations performed? Given that MnO2 can exhibit magnetic properties, it is important to clarify whether spin polarization was considered and how it affected the results.

R/ We are embarrassed, because we forgot to include that information; we have corrected the mistake and added:

All calculations were performed with spin polarization.

However, we did not include DOS nor band structure, because we weren’t interested in the electronic properties of the monolayer + heavy metal, since they are toxic for the environment and for the people handling it, so the effect of spin polarization on these calculations won’t affect the structural and energetic results.

**Regarding Results and Discussion:**

1. The adsorption energies indicate a strong binding of Pb compared to Cd. Could the authors provide a more in-depth analysis of the electronic structure changes upon adsorption to explain this difference? For example, a comparison of the density of states (DOS) or partial density of states (PDOS) for Cd and Pb adsorption would be helpful in section 3.2.1. Structural properties of the adsorption of Cd and Pb on the 1T-MnO2 monolayer.

R/ We have added new paragraphs and new figures in order to expand on the changes produced by Cb and Pb in the monolayer. However, as we mentioned in the previous comment, investigating changes in the electronic properties is not one of our objectives.

2. The Bader charge analysis suggests ionic bonding. Could the authors supplement this analysis with a discussion of the bond lengths and bond angles between the adsorbed atoms (Cd and Pb) and the substrate atoms (Mn and O)? This would provide further evidence to support the nature of the bonding.

R/ While there exists a general tendency for the chemical adsorption to indicate covalent (polar or non-polar) or ionic bonds, and the physical adsorption implies weaker van de Waals forces, there are exceptions. An analysis of the Bader charge together with the difference in charge, which shows the transference and the distribution of the charge of the interaction between the atomic adsorbates, the surface, and the atoms belonging to it, affords a more direct and reliable way for determining the type of bond.

Figures 10 and 11 reveal the dynamics of the charge transfer when the Cd or the Pb are adsorbed at site PMn. In both cases, the charge is donated by the adsorbate mainly to the first and second neighbors within the monolayer. The O4, O5, and O11 oxygen atoms (first neighbors) and the Mn5 atom exhibit a more significant gain of charge, which corresponds to the regions of intense yellow visible in the figure.

In particular, the charge redistribution represented in Figures 10 and 11 underlines the hybrid nature of the bond. A blue surface (charge depletion) involves both adsorbates, which is reminiscent of an ionic bond. Nevertheless, within the substrate itself (the monolayer), the charge gain in the oxygen atoms is manifested as yellow rings in its peripheries, markedly contrasting with the blue zones in their centers. This visual pattern signifies a local charge redistribution within the monolayer. This redistribution, intrinsic to the structure of the substrate, is a distinctive characteristic of interactions with an emerging polar covalent component, going further than a purely ionic behavior.

This behavior is exemplified by the adsorption of Pb (Fig. 11). Here, the charge donated by the Pb is distributed equally between the six neighboring oxygen atoms at the adsorption site (first neighbors O4, O5, and O11, and second neighbors O1, O7, and O8, shown in Fig. 7) They coincide with the six distinct yellow spherical regions that surround the site in Figure 11. Simultaneously, the Mn5 atom, just below the adsorbate, gains the greatest quantity of charge, which corresponds to the small yellow zones around it (see Fig. 11). The efficiency and reach of this charge redistribution mechanism are key factors that explain the high sensitiveness of the IT-MnO_2_ monolayer to the processes of adsorption of Cd and Pb.

Although the difference in the electronegativity (ΔEN) suggests a predominantly ionic character for Cd–O (ΔEN = 1.75) and a polar covalent character for Pb–O (ΔEN = 1.11), analysis of the Bader charge distribution reveals a more complex behavior. The transferred charges (~1.0 |e⁻| for Cd and ~1.4 |e⁻| for Pb) quantitatively satisfy the criterion of classical ionization. However, this charge donated by the metals is not localized in individual oxygen atoms, but rather is delocalized among the six oxygen atoms of the substrate (first and second neighbors). This redistribution adopts an extended lattice pattern that goes beyond the immediate neighbors and is stabilized by the structural disposition O_superior_–Mn–O_inferior_ (Fig. 4). This delocalization contradicts the expected localized geometry for discrete ionic bonds. As a consequence, the sorbent exhibits an emerging covalent character, although the ionic character predominates. The charge excess is accumulated mostly in the bond regions, and not in the oxygen nuclei—a phenomenon observed in both adsorbates.

3. The manuscript mentions that the 1T-MnO2 monolayer is "easily exfoliable." How does the calculated exfoliation energy compare to other known 2D materials? Providing a comparative context would strengthen this claim.

R/ In order to compare the value of the exfoliation energy obtained in this investigation for the 1T-MnO2 monolayer with the exfoliation energies of monolayers of other materials that are considered easily exfoliable, we have added the following text:

Additionally, the value 16.925 meV/Å for 1T-MnO2 is close to reported values of the exfoliation energy for monolayers of other materials, such as VO_2_ (16.250 meV/Å), CrO_2_ (18.690 meV/Å), MoO_2_ (19.210 meV/Å) [47], MgBr_2_ (17.262 meV/Å), MgI_2_ (15.808 meV/Å), MoS_2_ (18.211 meV/Å), and VS_2_ (17.071 meV/Å) [48], monolayers that are classified as easily exfoliable.

4. While the pristine 1T-MnO2 monolayer's stability is discussed, how does the adsorption of Cd and Pb affect the stability of the overall system? Were any calculations performed to assess the stability of the Cd/MnO2 and Pb/MnO2 systems (e.g., phonon dispersion calculations for the adsorbed systems)?

R/ We did not carry out calculations to evaluate the dynamic stability of the Cd/MnO2 and Pb/MnO_2_ systems, but we did do it for the pristine IT-MnO_2_ monolayer. In the majority of our published articles, we chose pristine systems because of their properties and applications, as well as their performance and reliability. The main goal of our investigation was to evaluate the system’s ability to capture and the intensity of the adsorption strength (physical or chemical adsorption). Our future interest will lie in the efficacy of the material for immobilizing the toxic ions of a solution, an act that in itself will mitigate the immediate environmental risk on avoiding its dispersion. Once the metal is trapped in the monolayer, the proposed strategy is to handle or dispose of all of the solid (the adsorbate-adsorbent) in a controlled manner. Therefore, the long-term stability of the metal-surface complex, although interesting from the scientific point of view, is not within the scope of interest of this study, since it does not change the ultimate purpose of capture and confinement.

5. The conclusion mentions that the pristine monolayer exhibits semiconductor behavior. How does the adsorption of Cd and Pb affect the electronic properties of the 1T-MnO2 monolayer (e.g., changes in the band gap, metallization)? This is crucial for understanding the potential applications of this material in heavy metal removal.

.R/ The principal aim of this investigation was to capture and retain the toxic heavy metal atoms Cd and Pb, i.e. to evaluate the efficacy of a MnO_2_ monolayer in the adsorption of heavy and toxic metallic ions. At the same time, we sought to quantify the intensity of the strength of the adsorbate/substrate interaction (physical or chemical adsorption) by means of the calculation of the energy of adsorption, as well as to determine whether or not the material had the capacity to be reused, which is crucial for its potential practical application.

The focus of the study centered on the capture and retention of these atomic adsorbates, since many of these metals represent a significant risk for human health and ecosystems on forming highly contaminating species. Their presence in agricultural aquifers and soils is a widespread environmental problem. The strategy of immobilizing them on a solid surface like a MnO_2_ monolayer offers a promising solution for their stable confinement, enormously facilitating their safe handling and controlled final disposition.

While we are conscious of the fact that industry prioritizes the use of safe and sustainable materials, the current phase of our research is limited to supplying the scientific bases that validate its efficacy as a reliable adsorbent. The possible applications or later development are not within the scope of this specific study.

**Regarding Broader Context and Implications:**

6. How do these calculated adsorption energies compare to experimental values (if available) or to other theoretical studies on similar materials? A comparison with existing literature would help to validate the accuracy of the calculations.

R/ On doing a review of the calculated values for the adsorption energy in similar theoretical studies, we concluded that a direct comparison of the adsorption energy values with those calculated in the present investigation is difficult, for various reasons: They are expressed in different units, as for example KJ/mol or meV, while the energy calculated in our investigation is in J. But what most makes a comparison difficult is that other researchers use calculation methods with different approximations. In spite of this difficulty, the reliability of the calculations is guaranteed when comparing the structural parameter values of the pristine monolayer with those reported by other authors, since they are similar even though they used different approximations from those in our investigation.

7. What are the potential limitations of this study? For example, the calculations are performed for an ideal 1T-MnO2 monolayer in vacuum. How might the presence of defects, surface functionalization, or a solvent environment affect the adsorption behavior in real-world applications?

R/ This study involves some restrictions, since the calculations were carried out for an ideal monolayer of IT-MnO_2_ in a vacuum and in base state (T → 0 K, P ≈ ±1 kbar), a setting that differs a little from the experimental conditions where this type of material could exhibit some defects, surface functionalization, or a solvent environment. Although the idealization of the system allows an exhaustive, controlled analysis, we recognize that:

- **Structural defects** (vacancies and impurities) can significantly modify some of the structural or electronic properties of the T-MnO_2_ monolayer system, which could have an impact on the adsorption of atomic or molecular adsorbates. The first part of this statement was reported by us in a preceding article, in which the effects of monovacancies of oxygen and manganese on the properties of a T-MnO_2_ monolayer grown on graphene were analyzed [4]. Likewise, in this investigation it is conjectured that the oxygen vacancies *could improve* the adsorption capacity of mercury and species of mercury on the T-MnO_2_/graphene heterobilayer. This conjecture was motivated by another article we published entitled “Adsorption and disassociation of species of mercury on the surface IT-MnO_2_/graphene” [5].
- **Environmental effects** (solvents and humidity) were not taken into account because of the computational complexity of modelling solid-liquid interfaces. Previous related studies such as “Synthesis and structural properties of MnO2 as adsorbent for the removal of lead (Pb^2+)^ from aqueous solution” [6] show that the samples exhibit excellent efficiency in the elimination of Pb^2+^ from aqueous solutions. The maximum elimination of lead ions from the aqueous solution reaches 99.8%. Furthermore, the kinetic study determined that the adsorption of lead ions in the prepared samples is produced through the process of chemisorption. Therefore, α-MnO₂ can be used as a possible adsorbent of metallic lead ions.

In spite of the restrictions involved in our calculations, the results obtained in this investigation afford us valuable and reliable information on the mechanisms of the adsorption of toxic heavy metal atoms onto the T-MnO_2_ monolayer. The good agreement of our results with the existing experimental results [6], in particular related to the type of adsorption that occurs in both Pb/MnO_2_ systems, i.e. chemical adsorption, supports the reliability of the calculations, their relevance, and their prospective applications in environmental remediation, although in future studies we should explicitly explore defects, functionalization, and the effects of solvents in order to close the gap between ideal models and more realistic models.

8. What are the implications of these findings for the design of materials for heavy metal removal? Could the authors discuss the potential advantages and disadvantages of using 1T-MnO2 monolayers compared to other materials?

R/ The implications of this study for the design of materials for environmental remediation are centered on the potential of IT-MnO_2_ monolayers as selective adsorbents for heavy metals. While bidimensional materials present challenges for synthetic scalability with regard to more economical systems in volume, their value resides in applications of micro-technology where the extreme surface-volume relation (∼10^2^ greater than 3D) allows developing miniaturized devices for the purification of aqueous bodies with unprecedented efficiency.

The specific choice of IT-MnO_2_ was based on critical advantages:

1. Environmental viability: Among the transition metal oxides, MnO_2_ is an important semiconductor type n that is characterized by its great natural abundance, low cost, and lack of toxicity, as well as by its attractive physio-chemical properties [7–12].
2. Controlled synthesis: Possible compatibility with scalable methods such as chemical vapor deposition (CVD), which allows obtaining monolayers with low defect density [13,14].
3. Specialized active sites: The IT phase generates unique adsorption geometries (P_Mn_ sites) where the surrounding oxygen atoms, with high electronegativity, (χ = 3.44), create “electrostatic traps” ideal for metallic ions. This optimizes three key aspects:

- Ability to capture (adsorption energy >3.0 eV for Pb^2+^)
- Facility of extraction (relatively low desorption energy for captured Cd^2+^ ions)
- Ease of subsequent handling (chemical stability that allows safe disposal of Pb^2+^ ions).

Compared with traditional adsorbents such as zeolites and activated carbon, IT-MnO_2_ offers greater intrinsic selectivity and mechanical durability. However, its industrial implementation requires advances in:

- Techniques of transference of monolayer to flexible substrates.
- Strategies of regeneration to overcome its disposable nature.

9. I will recommend authors to use (https://doi.org/10.1093/narmme/ugae005 and https://doi.org/10.1021/acsmedchemlett.3c00537 and https://doi.org/10.3390/scipharm93010006 references in introduction.

.

R/ Thank you for the suggestion, but on reviewing the articles, we found that in spite of the fact that there areadsorption, they are not about dichalcogenide monolayers, and we were not able to find a close relation to the material and the atoms that are adsorbed on the monolayer of the investigation carried out in this study.

Referencias

[1]	A. Stroppa and G. Kresse, “The shortcomings of semi-local and hybrid functionals: what we can learn from surface science studies,” *New J Phys*, vol. 10, no. 6, p. 063020, Jun. 2008, doi: 10.1088/1367-2630/10/6/063020.

[2]	M. Capdevila-Cortada, Z. Łodziana, and N. López, “Performance of DFT+U approaches in the study of catalytic materials,” *ACS Catal*, vol. 6, no. 12, pp. 8370–8379, Nov. 2016, doi: 10.1021/ACSCATAL.6B01907/ASSET/IMAGES/LARGE/CS-2016-01907W_0005.JPEG.

[3]	S. Swathilakshmi, R. Devi, and G. Sai Gautam, “Performance of the r2SCAN Functional in Transition Metal Oxides,” *J Chem Theory Comput*, vol. 19, no. 13, pp. 4202–4215, Jul. 2023, doi: 10.1021/ACS.JCTC.3C00030/SUPPL_FILE/CT3C00030_SI_001.PDF.

[4]	J. D. Morinson-Negrete, C. Ortega-López, and M. J. Espitia-Rico, “Effects of Mono-Vacancies of Oxygen and Manganese on the Properties of the MnO2/Graphene Heterostructure,” *Materials 2022, Vol. 15, Page 2731*, vol. 15, no. 8, p. 2731, Apr. 2022, doi: 10.3390/MA15082731.

[5]	J. D. Morinson Negrete, G. C. Jiménez, J. F. Murillo García, C. O. López, and M. J. Espitia Rico, “Adsorption and dissociation of mercury species on the surface of 1T-MnO2/graphene,” *Computational Condensed Matter*, vol. 32, p. e00704, Sep. 2022, doi: 10.1016/J.COCOM.2022.E00704.

[6]	S. T. El-Wakeel, R. S. El-Tawil, H. A. M. Abuzeid, A. E. Abdel-Ghany, and A. M. Hashem, “Synthesis and structural properties of MnO2 as adsorbent for the removal of lead (Pb2+) from aqueous solution,” *J Taiwan Inst Chem Eng*, vol. 72, pp. 95–103, Mar. 2017, doi: 10.1016/J.JTICE.2017.01.008.

[7]	D. Barreca, A. Gasparotto, F. Gri, E. Comini, and C. Maccato, “Plasma-Assisted Growth of β-MnO2 Nanosystems as Gas Sensors for Safety and Food Industry Applications,” *Adv Mater Interfaces*, vol. 5, no. 23, p. 1800792, Dec. 2018, doi: 10.1002/ADMI.201800792;JOURNAL:JOURNAL:21967350;WGROUP:STRING:PUBLICATION.

[8]	L. Bigiani *et al.*, “Hydrogen Gas Sensing Performances of p-Type Mn3O4 Nanosystems: The Role of Built-in Mn3O4/Ag and Mn3O4/SnO2 Junctions,” *Nanomaterials 2020, Vol. 10, Page 511*, vol. 10, no. 3, p. 511, Mar. 2020, doi: 10.3390/NANO10030511.

[9]	S. Mallakpour, A. Abdolmaleki, and H. Tabebordbar, “Employment of ultrasonic irradiation for production of poly(vinyl pyrrolidone)/modified alpha manganese dioxide nanocomposites: Morphology, thermal and optical characterization,” *Ultrason Sonochem*, vol. 41, pp. 163–171, Mar. 2018, doi: 10.1016/J.ULTSONCH.2017.09.036.

[10]	S. Balamurugan, A. Rajalakshmi, and D. Balamurugan, “Acetaldehyde sensing property of spray deposited β-MnO2 thin films,” *J Alloys Compd*, vol. 650, pp. 863–870, Nov. 2015, doi: 10.1016/J.JALLCOM.2015.08.063.

[11]	W. Zhang, C. Zeng, M. Kong, Y. Pan, and Z. Yang, “Water-evaporation-induced self-assembly of α-MnO2 hierarchical hollow nanospheres and their applications in ammonia gas sensing,” *Sens Actuators B Chem*, vol. 162, no. 1, pp. 292–299, Feb. 2012, doi: 10.1016/J.SNB.2011.12.080.

[12]	P. V. Shinde *et al.*, “Hydrothermally grown α-MnO2 interlocked mesoporous micro-cubes of several nanocrystals as selective and sensitive nitrogen dioxide chemoresistive gas sensors,” *Appl Surf Sci*, vol. 442, pp. 178–184, Jun. 2018, doi: 10.1016/J.APSUSC.2018.02.144.

[13]	L. Bigiani, C. Maccato, A. Gasparotto, C. Sada, E. Bontempi, and D. Barreca, “Plasma-Assisted Chemical Vapor Deposition of F-Doped MnO2 Nanostructures on Single Crystal Substrates,” *Nanomaterials 2020, Vol. 10, Page 1335*, vol. 10, no. 7, p. 1335, Jul. 2020, doi: 10.3390/NANO10071335.

[14]	Y. O. Yesilbag, F. N. Tuzluca Yesilbag, A. Huseyin, A. J. S. Salih, and M. Ertugrul, “MnO2 nanosheets synthesized on nitrogen-doped vertically aligned carbon nanotubes as a supercapacitor electrode material,” *J Alloys Compd*, vol. 925, p. 166570, Dec. 2022, doi: 10.1016/J.JALLCOM.2022.166570.
